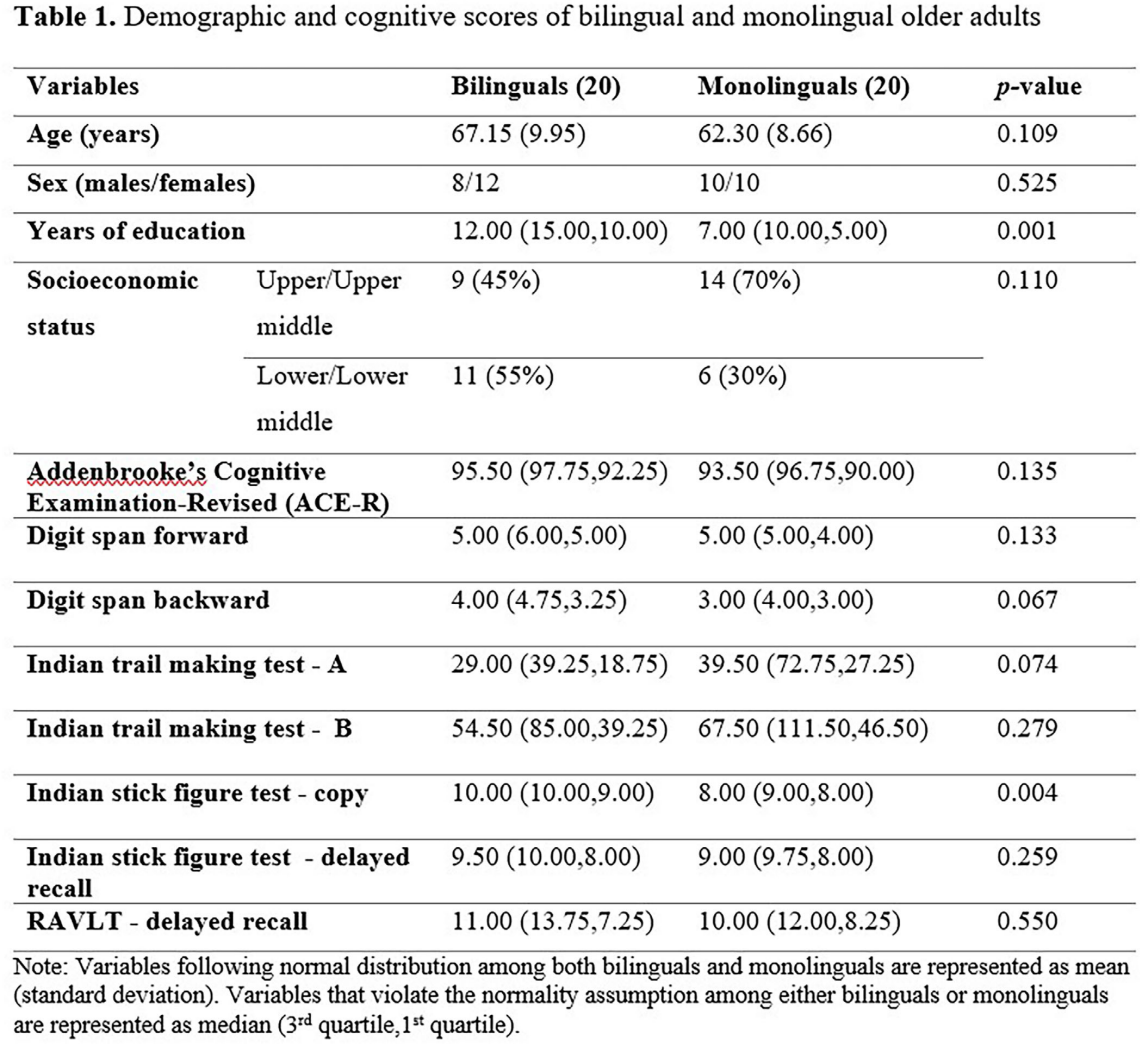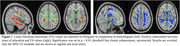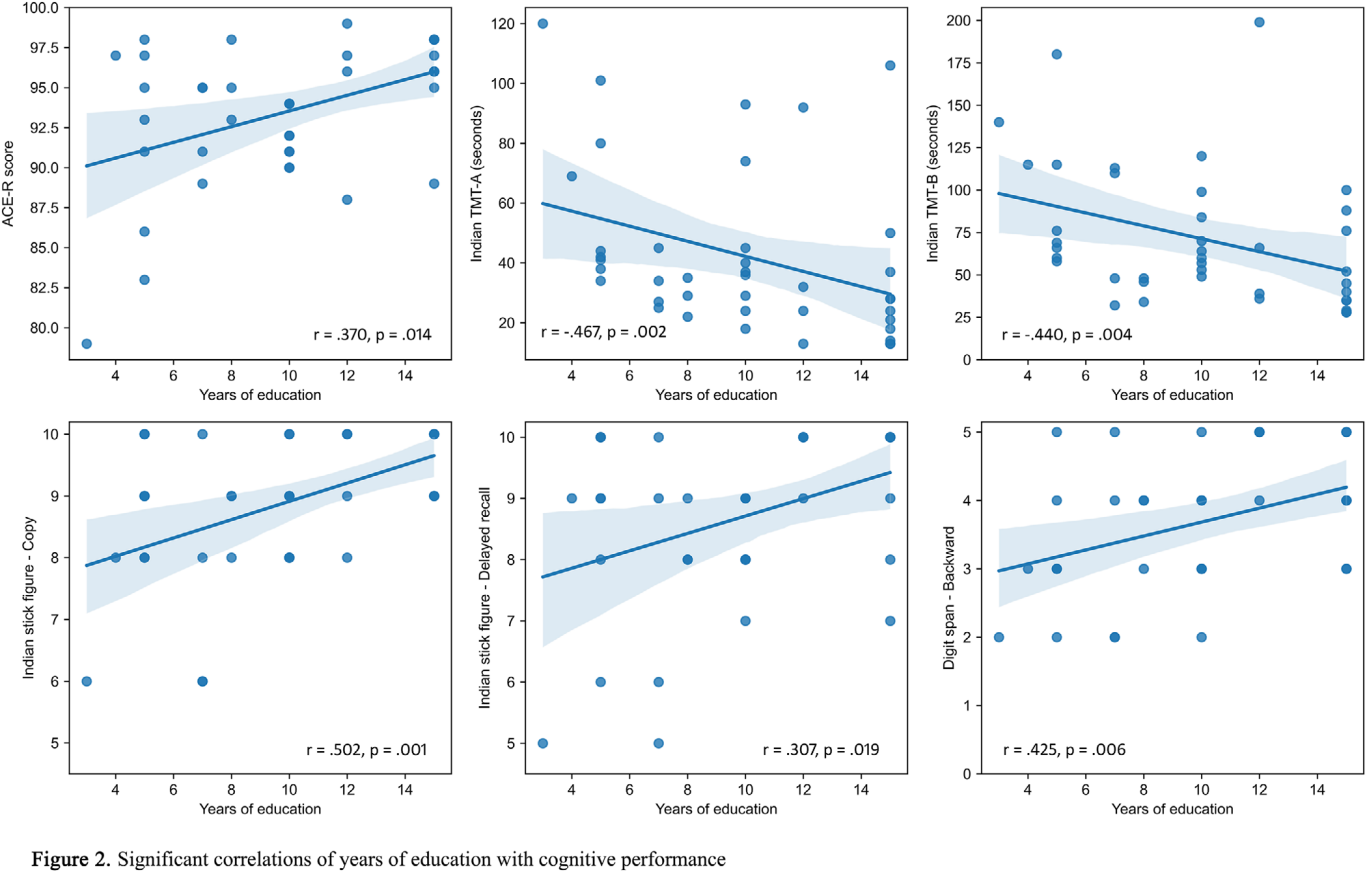# Bilingualism and education may have distinct reserve/resilience trajectories: Evidence from white matter changes

**DOI:** 10.1002/alz.090677

**Published:** 2025-01-03

**Authors:** Nithin Thanissery, Raghavendra Kenchaiah, Shailaja Mekala, Sireesha Jala, Sunil Kumar Khokhar, Amulya Rajan, Vani Kashyap, Bapiraju Surampudi, Subhash Kaul, Suvarna Alladi

**Affiliations:** ^1^ National Institute of Mental Health and Neuro Sciences, Bengaluru, Karnataka India; ^2^ Nizam’s Institute of Medical Sciences, Hyderabad, Telangana India; ^3^ International Institute of Information Technology, Hyderabad, Telangana India

## Abstract

**Background:**

Bilingualism and education have been known to enhance reserve/resilience through white matter (WM) microstructural changes. Reserve/resilience is the ability of the brain to cope with neurocognitive adversities. Two key hypotheses have been proposed under this framework. Firstly, brain reserve, which refers to the structural integrity of the brain to preserve cognitive functioning. Secondly, cognitive reserve, which suggests that cognitive functioning can be maintained despite reduced structural brain integrity. Notably, the concepts of brain reserve and cognitive reserve exist along a continuum of brain health. Given the criticism surrounding bilingual literature due to the potential influence of education, the present study aims to delineate the effects of bilingualism and education (proxy measures of reserve/resilience) on WM integrity.

**Method:**

40 cognitively normal older adults (20 bilinguals and 20 monolinguals) were recruited. Cognitive evaluation was carried out using a set of assessments/tests (see Table 1). Diffusion tensor imaging was done using a 3‐Tesla scanner. Tract‐based spatial statistics was performed. Fractional anisotropy (FA), axial diffusivity, radial diffusivity, and mean diffusivity were assessed.

**Result:**

Bilinguals did not differ from monolinguals in age, sex, and socioeconomic status (Table 1). Bilinguals were more educated than monolinguals. Bilingual older adults had lesser WM integrity (i.e., lesser FA values) compared to monolinguals in the regions that adapt to bilingual experience as well as those that are affected by aging (figure 1). Despite having lesser FA, bilinguals had similar cognitive functioning compared to monolinguals (Table 1). Contrastingly, higher education was associated to higher FA values in several commissural and association WM fibres (figure 1). Education also showed a positive relationship with cognitive scores (figure 2).

**Conclusion:**

The study demonstrate that bilingualism is associated with better cognitive reserve (preserved cognition despite reduced structural integrity), while education is associated with better brain reserve (better structural integrity and cognition). Advances in reserve/resilience theory propose that the neuroprotective effect, which is responsible for better structure (brain reserve) will be followed by a compensatory effect (cognitive reserve) as the structural integrity decline over time. Our study suggests that bilingualism and education may have two distinct reserve/resilience trajectories in the face of age‐related changes.